# Is chewing khat associated with mental health disorders? A scoping review of the content and quality of the current evidence base

**DOI:** 10.1186/s13011-023-00545-y

**Published:** 2023-06-27

**Authors:** Amanti Baru Olani, Mulusew Gerbaba, Masrie Getnet, Matiwos Soboka, Tom Decorte

**Affiliations:** 1 Institute for Social Drug Research, Department of Criminology, Criminal Law and Social Law, Ghent University, Universiteitstraat 4, Ghent, 9000 Belgium; 2 Department of Sociology, Jimma University, Jimma, P.O.Box 378 Ethiopia; 3 Department of Epidemiology, Institute of Health, Jimma University, Jimma, P.O.Box 378 Ethiopia; 4 Department of Psychiatry, Institute of Health, Jimma University, Jimma, P.O.Box 378 Ethiopia

**Keywords:** Khat, Catha edulis, Mental health disorders, Psychological distress, Scoping review

## Abstract

**Background:**

Khat (*Catha edulis*) is a plant commonly found in the horn of Africa whose leaves are chewed for their psycho-stimulant effects. Several studies have demonstrated the association between khat use and mental health problems. Nevertheless, evidence is mixed and inconsistent, warranting further review of available studies. This scoping review is aimed at investigating the content and quality of evidence base on the associations between khat use and mental health disorders and suggesting avenues for further research.

**Methods:**

We used a scoping review methodology to map the existing evidence using PubMed, SCOPUS, Embase, and CINAHL databases. Primary studies focusing on the association between any pattern of khat use and any form of mental health disorders are included. The review focused on all age groups, any study design, all geographical locations, and any publication year. The terms used for searching eligible studies include khat, mental disorders, and various alternative terminologies. Narrative review is employed to present findings.

**Results:**

7,121 articles were found, of which 108 were eligible, conducted across 12 different countries. The majority of the studies was done during the last ten years and the studies mostly employed cross-sectional design. About 10 different categories of mental health disorders have been identified as showing associations with khat use. Despite many contradictory findings between the studies, most of the evidence base suggests that khat use is associated with mental health disorders. Non-specific psychological distress is the most frequently mentioned mental health problem (reported in 26.9% of the studies). Khat use as a predictor variable is mostly assessed using a ‘yes/no’ category, and as a result, *dose-dependent effects* of khat use on mental health are not given much consideration.

**Conclusion:**

Although most of the studies associate khat use with mental health disorders, the causal relationships are inconclusive given the cross-sectional design of the studies, and the presence of potential confounders and several forms of biases. Available studies also report contradictory findings. Further studies are recommended using prospective designs, standardized and valid measures of khat use, and focusing on specific types of mental health disorders.

**Supplementary Information:**

The online version contains supplementary material available at 10.1186/s13011-023-00545-y.

## Background

Many researchers believe that khat - one of the most popular, and often unrestricted, psychoactive substances within low-income countries in East Africa and the Arabian Peninsula - may be a cause of mental health problems [1–7]. Khat leaves contain *cathine* and *cathinone*, both structurally related to amphetamine [8]. Its effects on the chewer include a boost of energy, excitement, and sociability while chewing [9], followed by anxiety, irritability, and sleep problems [8].

The consumption of khat is a deeply rooted socio-cultural tradition in countries like Ethiopia, Somalia, Kenya and Yemen [10]. The chewing of the plant’s tender leaves and shoots dates to ancient times as a means to facilitate social interaction and for religious purposes, and these practices persist in modern times. Additionally, farmers and other laborers use it to alleviate physical exhaustion, while vehicle drivers and students use it to enhance their focus [11]. Determining the precise prevalence of khat chewing is challenging due to its usage continuing to evolve rapidly, as the use of khat has expanded beyond its historical social and geographic circles. The existing evidence is also fragmented due to little effort made to conduct large-scale studies. A closer look at some of the existing literature, however, reveals that a quite significant proportion of different population groups chew khat mostly in the traditional use countries in the East Africa and the Arabian Peninsula. A meta-analysis of 18 studies, for instance, found that the pooled prevalence of khat chewing among youth students in Ethiopia was 16.7% [12]. In the southwestern Uganda, a study conducted on three occupational groups reveals that 20.4% of the respondents use khat at the time of the study, and 31.5% were ever chewers of khat [5]. Among armed personnel in Somalia, 36.4% prevalence of khat use in the week before the interview was reported [6]. In the capital city of Yemeni, Sana’a, 86% of the males and 50% of the females were khat chewers, and the majority of them were between the ages of 15 and 30 years [7]. The use of khat and the potential socioeconomic and health hazards it poses have also been a cause for increasing concern in various regions around the world, including Europe, North America, Canada, and Australia mainly as a result of immigrants from traditional use regions who bring with them the custom of chewing khat [13–15].

To this date, the potential mental health risks, and benefits of khat chewing are sources of increasing policy and academic debates. In 1985, cathinone and cathine were evaluated for control under the Convention of Psychotropic Substances and recommended to be placed in Schedule I and Schedule II, respectively [16]. Despite these developments, khat is not currently under international control, and different countries have their own policies regarding its use, which can range from criminalization to non-regulation.

Many studies focus on a possible association between excessive khat use and the occurrence of mental health disorders, such as psychosis [1, 17–21], depression, anxiety, stress [22, 23], suicidal attempts [24], mental distress [25], and paranoid ideation [26]. On the other hand, other studies did not find a significant association between khat use and mental health disorders [27, 28]. It is within this context that this scoping review was conceived to summarize and assess the evidence on the association between khat use and mental health disorders. Thus far, two systematic reviews have been conducted to assess the mental health effects of khat chewing. A meta-analysis of 35 studies showed that the odds of psychiatric symptoms among people who use khat were 2.22 times higher than the odds among people who do not use khat [2]. Similarly, a meta-analysis of six primary studies among Ethiopian college students indicates that students who use khat were 2.01 times more exposed to common mental disorders than those who do not use [3]. However, evidence from non-quantitative studies was not summarized and efforts to encapsulate the nature of the studies (e.g., designs and measurement issues) did not get sufficient attention. As a result, this scoping review was conducted:

1. To summarize the association between mental health issues and khat use.
2. To characterize the relevant studies in terms of geographical area, date of publication, and study design.
3. To identify limitations in the evidence base that need further study.

## Methods

### Protocol design & procedure

We conducted a scoping review to map and summarize the evidence available on the association between mental health disorders and khat use, and identify limitations in the existing body of research. We followed the five stages specified in the methodology of a scoping review as described by Arksey and O’Malley [29]. These are: (1) identifying the research question(s); (2) identifying potentially relevant studies; (3) selecting eligible studies; (4) charting the data; (5) collating, summarizing, and reporting the results [29].

### Stage 1: identify the research question

The main research question that guided this scoping review is “what is known about the association between khat use and mental health disorders?” It is true that the term mental health disorders is a very broad concept and search results could be unmanageably high. However, a broad research question is recommended as it may reduce the likelihood of missing relevant articles [29]. Once the breadth of existing literature and the number of bibliographic references is identified, a decision of whether it is important to revise the scope of the research question is determined. In this study, the research question is maintained up to the end since the objective was to present a comprehensive picture of the general relation between the use of khat and mental health disorders.

### Stage 2: identifying relevant studies

- Eligibility criteria.

The following inclusion and exclusion criteria were utilized to guide the search and selection of review articles.

**Table:** 

Inclusion criteria		Exclusion criteria
**Population**	• All age groups and both sexes of participants. • Research that looks at the general population, as well as at specific population groups.	• Opinions, letters to the editor, magazine, and newspaper articles. • Studies where khat use is generally reported as a substance use without clarification. • Published in other languages than English. • Articles not based on empirical data.
**Concept**	• Research articles should examine the link between khat use and mental health disorders. • The articles should be based on empirical studies. • Any pattern of khat use. • Any mental health conditions. • Published primary studies as well as primary grey literature including unpublished thesis/dissertations and conference proceedings. • Any study designs. • Both quantitative and qualitative study approaches.	
**Context**	• All geographical locations. • Any publication year.

b. Search strategy and databases.

A comprehensive literature search was conducted in four databases to search for articles that fulfill the eligibility criteria: PubMed, SCOPUS, Embase, and CINAHL. A secondary search was performed in Google Scholar to identify articles that are not indexed in the databases listed above. In addition, hand searches were conducted on Google and OpenGrey to identify unpublished literature.

The following terms were used to search for articles from the databases and search engines (“Catha“[Mesh] *OR “Catha edulis”*[tw] OR khat[tw] OR Murungi[tw] OR Miraa[tw] OR Chat[tw] OR Qat [tw]) AND (“Mental Disorders“[Mesh] OR “Mental health” [tw] OR “mental illness” [tw] OR “mental disorder*” [tw] OR “mental problem*”[tw] OR “mental distress” [tw] OR psycho*[tw] OR psychiatr*[tw]). Finally, references were extracted and imported to the EndNote X9 reference management system. Search terms addressing individual mental health disorders - e.g. depression - were not included to ensure the number of studies generated was realistic for the time frame of the review.

### Stage 3: study selection

The study selection procedure followed are described as follows:

- Titles and abstracts identified by the search strategy were evaluated against the eligibility criteria by two of the authors (ABO and MS).
- After title and abstract review, full texts of included articles were read.
- Following this, full texts sourced for all articles meeting the inclusion criteria were used for analysis. A PRISMA flow diagram was used to report the whole search process.

### Stage 4: charting the data

Eligible studies were charted using data extraction fields presented in the JBI guidance for conducting systematic scoping reviews [30] and other fields relevant to the research questions are added (i.e., study design, predictor variable and its measurement, and limitations of the studies and their recommendations).

1. Author (s).
2. Year of publication.
3. Origin (where the study was conducted).
4. Aims/purpose.
5. Study population and sample size.
6. Study design.
7. Outcome.
8. Predictor variable and its measurement.
9. Key findings that relate to the scoping review research questions.
10. Limitations of the studies and their recommendations.

An excel data extraction form was used to obtain details about the above information.

### Stage 5: collating, summarizing, and reporting the results

The results are summarized both quantitatively and qualitatively to give an account of the collected data.

1. A quantitative analysis is used to map the studies in tabular and diagrammatic form, showing the distribution of studies by theme, period of publication, country of origin, and study design.
2. A qualitative summary is used to provide a thematically organized narrative synthesis describing how the studies identified relate to the research questions of this review and the main findings emerging from these studies.

## Results

### Study selection

**Fig. 1:**
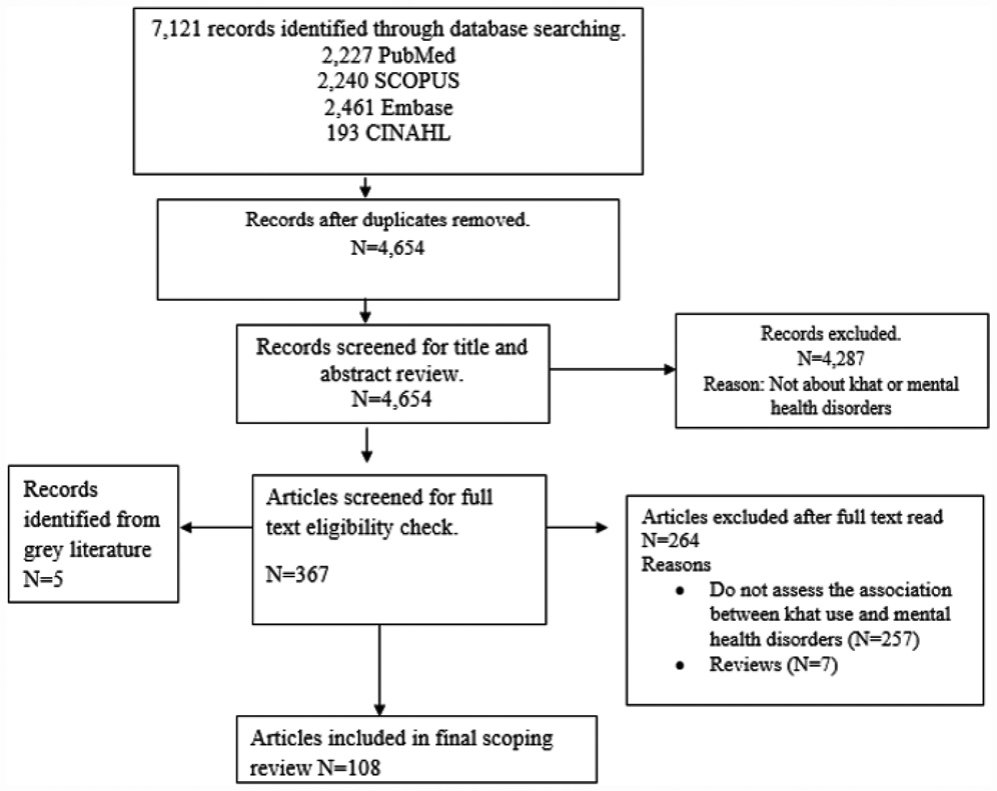
Flow diagram of screening process (PRISMA)

The initial online search of four databases (PubMed, SCOPUS, Embase, and CINAHL) from 07 to 12 August 2021 yielded a total of 7,121 articles. After the removal of duplicates using EndNote X9 software, the number of articles was reduced to 4,654. The titles and abstracts of these 4,654 articles were then reviewed, and 4,287 articles were excluded because they were not about khat or mental health disorders (see Fig. 1 below). The non-khat studies were generated because of including the term ‘chat’ in the search terms. The articles nominated for full-text eligibility check were 367, and from these, 264 articles were excluded after a full-text read because they are irrelevant as per the specified inclusion criteria (see methods section). The search of gray literature produced 5 eligible articles, and finally, N = 108 articles (see supplementary 1) are included in this scoping review.

### Geography of the included studies

The articles included in this scoping review are from 12 different countries. Half of the included studies (50%) are from Ethiopia, 10.2% from Yemen, and 8.3% from Saudi Arabia. These are the countries where khat has been traditionally consumed on a regular basis by a significant number of people. Almost one-third of the studies (31.5%) focused on 9 other countries. While khat is part of a long-established tradition in Kenya, Somalia and Somaliland, its use in Australia, Germany, Israel, the Netherlands, the UK, and the US is mostly among immigrants from the traditional khat-chewing countries in East Africa and the Arabian Peninsula.

**Table 1:** Geographical distribution of the studies (n = 108)

Country		Number of studies		Percentage of studies	
Australia	5		4.6		
Ethiopia	54		50.0		
Germany	3		2.8		
Israel	1		0.9		
Kenya	5		4.6		
Saudi Arabia	9		8.3		
Somalia	4		3.7		
Somaliland	2		1.9		
the Netherlands	4		3.7		
UK	7		6.5		
USA	3		2.8		
Yemen	11		10.2		
**Total**	**108**		**100.0**		

### Study designs

The studies included are of considerable variety in terms of designs. The majority of the studies (71.3%%) used a cross-sectional design. Within these cross-sectional studies, an institution-based design (health facilities or school-based studies) takes the largest share (38.9%), a community-based cross-sectional study accounts for 29.6% of the studies and lastly, 2.8% are mixed method (cross-sectional and qualitative) studies.

**Table 2:** Study designs (n = 108)

Designs	Frequency	Percent
Case report/ Case series	14	13.0
Case-control	3	2.8
Community-based cross-sectional study	32	29.6
Institution-based cross-sectional study	42	38.9
Mixed method (cross-sectional + qualitative)	3	2.8
Prospective Cohort	4	3.7
Qualitative interviews	6	5.6
Randomized Controlled Trial (RCT)	1	0.9
Retrospective cohort	1	0.9
Single arm pre-post study design	2	1.9
**Total**	**108**	**100.0**

### Year of publication

The first study assessing the link between mental health disorders and khat use identified in this review was published in 1981 by Dhadphale and Mengech [31]: a case report of ‘Miraa (*Catha edulis*) as a cause of psychosis’. There seems to have been a stagnation in the number of studies interested in the topic for the first three decades (1981–2010) and it was only starting from 2011 that a remarkable increase in the number of publications is observed. The period from 2011 to 2021 comprises 78.7% of the studies, indicating an increasing interest in the topic.

**Fig. 2:**
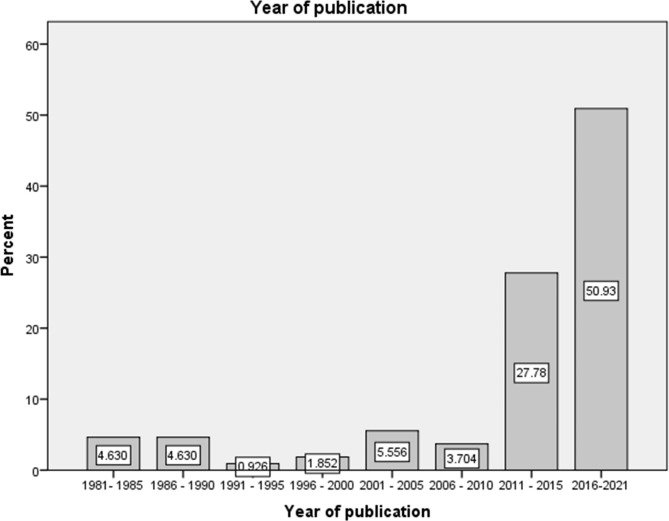
Year of publication

### Measurement of khat chewing patterns

After removing qualitative studies and case reports/case series (n = 22) due to their lack of rigorous focus on the measurement of khat consumption, we assessed how the remaining 86 studies measured khat use to examine its link with mental health disorders. About 70% of the studies measured khat chewing with a simple question about current or lifetime prevalence (‘Did you ever use khat? or Do you currently use khat’), with ‘yes/no’ as potential answers. The remaining studies employed tools like the modified version of “Alcohol Use Disorder and Associated Disabilities Interview Schedule-IV (AUDADIS-IV)”[32], a single Likert scale question with varying points (e.g., 2-point scale, 4-point scales) [33, 34], or questions about the age of the onset of chewing [35], and the amount of chewing [36]. Only two studies employed a standardized and khat-specific multi-item module for measuring khat chewing [37, 38].

### Mental health problems and related conditions associated with khat use

Of all the included articles, 100 (92.6%) of them used khat use as a predictor variable and the remaining 8 (7.4%) articles [39–46] considered khat use as an outcome variable. Ten different categories of mental health disorders associated with khat chewing are provided in Table 3 and the description of each identified association is provided in the subsequent sub-sections.

**Table 3:** Mental health disorders associated with khat chewing (n = 108)

Mental disorders and related conditions	Frequency	Percent
Depression, anxiety, stress	17	15.7
Non-specific (general) psychological distress	29	26.9
Insomnia	1	0.9
Mood status	4	3.7
Neurocognitive disorder	8	7.4
Psychopathy	2	1.8
Psychosis	17	15.7
Social phobia	1	0.9
Substance use disorder	16	14.9
Suicidal ideation	5	4.6
Other conditions related to mental health*	8	7.4
**Total**	**108**	**100.0**

*Other conditions related to mental health present in the evidence base include adherence and response to antipsychotic medication (n = 2, 1.9%), mental disorder induced suicide (n = 1, 0.9%), quality of life-mental health component (n = 4, 3.7%) and readmission for mental disorder treatment (n = 1, 0.9%)

A. Non-specific psychological distress.

Non-specific psychological distress is the most frequently mentioned mental health problem in connection with khat use, reported in 26.9% of the included studies. The evidence about this association is mixed, with some studies indicating the existence of significant associations and others reporting the absence of such associations [34, 47, 48][49]. The lowest reported Adjusted Odds Ratio (AOR) was 2.23 [50] and the highest AOR was 6.91 [51]. A hospital-based cross-sectional survey among patients with hypertension reported the absence of an association between psychological morbidity and khat chewing [48]. Some of the articles indicate that it is not khat in and of itself that is associated with non-specific psychological distress, but rather the frequency of use. For instance, people who use khat regularly or daily had eight times higher chance of experiencing mental distress than those who do not use khat (AOR = 8.122, 95% CI: 1.232–53.551), while people with more moderate use patterns (occasionally and one or more per week) have no significant chance of experiencing non-specific psychological distress than those who do not use khat [34]. One study found that ‘current use of khat’ is not associated with non-specific psychological distress, while life time prevalence of khat use is associated [52]. Qualitative findings, on the other hand, suggest that people use khat for the treatment of psychological distress [53].

B. Psychosis.

Of all the studies within the evidence base, 15.7% explore the association between psychosis and khat chewing. This makes psychosis the specific mental disorder frequently associated with khat chewing. Evaluation of the strength of this evidence shows that 64.7% (11 out of the 17 articles) relied on case report or case series designs, and 58.8% (10 out of the 17 articles) of the studies were conducted among migrants from traditionally khat chewing regions.

C. Depression, anxiety, and/ or stress.

Depression, anxiety and/or stress are grouped together in this review as several studies address these topics jointly. This category is reported by 15.7% of the articles included in this scoping review.

Several studies show a significant association [54–57], some studies report the absence of such association [28, 40, 41, 58] and one other study [59] reports the use of khat for alleviating depression and stress. In a cross-sectional study among university students, khat use was not associated with depression, anxiety, and stress in the univariate analysis [23]. Another studies among students found that khat chewing is insignificantly related to depression [40][41] and stress [41] after a multivariate logistic regression analysis.

On the other hand, there is also evidence showing that a history of khat chewing is significantly associated with depression, stress and anxiety. Prisoners who chew khat prior to incarceration were about two times more likely to develop depression (AOR = 2.47, 95% CI: 1.049–5.85, P = 0.039) than those who did not chew khat [57]. Among currently married women (15–49 years old) in rural Ethiopia, frequent khat use is also independently associated with depression (OR = 1.61) [54]. Among Jimma university staff (Ethiopia), khat chewing was associated with depression (AOR = 4.99, 95% CI: 2.57–9.69), anxiety (AOR = 2.94, 95% CI: 1.52–5.66) and stress (AOR = 2.78, 95% CI: 1.49–5.21) [55]. Another study shows that it is not just khat use which matters in causing anxiety and depression. The study revealed statistically significant differences in the levels of anxiety (F (2, 15) = 9.64, p < 0.05) and depression (F (2, 15) = 6.34, p < 0.05) among non-chewers, non-dependent khat chewers and dependent khat chewers. After conducting post-hoc multiple comparisons, dependent khat chewers scored significantly higher mean values in both anxiety and depression as compared to non-chewers and non-dependent khat chewers. Non-dependent khat chewers have almost the same mean score for anxiety and depression, as non-chewers [56].

The functional use of khat to relieve anxieties and depression have also been reported in a mixed method study [59].

D. Substance use disorder.

The fourth category of mental disorder associated with the use of khat is substance use disorder. About 15% of the included studies have reported such association. A measurement of khat dependence based on the Drug Abuse Screening Test-10 (DAST-10) among people who use khat shows that 2% reported no problems, and 17.3% reported a low level, 73.6% reported a moderate level and 7.1% reported a substantial level of dependence. The risk of being moderately dependent increases by 4.8 (95.0% CI 1.46–15.78) for daily chewers as compared to irregular chewers [35]. Assessment of khat use disorders among samples recruited from the general community and university students using DSM-5 criteria shows that 10.5% (95% CI: 7.9–13.9) were categorized as experiencing mild, 8.8% (95% CI: 6.4–12.0) moderate and 54.5% (95% CI 49.6–59.3) severe khat use disorders. Khat use disorder is prevalent among subjects with greater frequency and quantity of khat use [32].

The use of khat has also been associated with the prevalence of alcohol use disorders and nicotine dependence. Khat chewing was a predictor of alcohol use disorders (AOR = 5.11, 95% CI: 1.60, 16.33) among human immunodeficiency virus-infected patients attending an antiretroviral therapy clinic [60]. Chewing khat is one of the significant factors associated with an increased prevalence of alcohol use disorders (AOR = 3.26, CI:1.30, 8.15) among university undergraduate students [61]. Concerning nicotine dependence, the odds of nicotine dependence for people who use khat were three times higher (AOR 3.09, 95% CI:1.206–7.896) when compared to those who do not use khat among adult psychiatric patients [62]. Similarly, among people with a mental illness, there was a significant association between tobacco dependence and daily khat chewing (AOR = 13.51, 95% CI: 4.27, 42.74), chewing khat 2–3 times per week (AOR = 5.09, 95% CI = 1.37,18.95), or chewing khat once a week (AOR = 4.31, 95%CI: 1.04,17.78) [63].

E. Suicidal ideation.

Five studies have examined the association between khat use and suicidal ideation. The odds of suicidal ideation were higher among those having a history of khat use [64–66]. A cross-sectional study among khat-chewing mothers attending public health centers in Addis Ababa after child delivery shows that the odds of suicidal ideation were 8.48 higher among those who chew khat as compared to those who do not [66]. Similarly, a cross-sectional study on suicidal thoughts among university students in Ethiopia found that the odds of suicidal ideation range from 1.78 to 4.46 among khat-chewing students than their non-chewing counterparts [64, 65].

F. Neurocognitive disorder.

Neurocognitive disorders have been studied in 7.4% of the studies. People who chew khat scored less in tasks related to cognitive flexibility (which is the ability to adapt and restructure cognitive representations in response to changing situational demands) than those who do not chew khat [67]. Studies also found a negative effect of khat use on working memory [67, 68]. Although statistically significant [F(3, 57) = 3.98, p = 0.012], the extent of performance decline seen in people who use khat is modest [68]. On the other hand, no statistically significant difference [F(3, 57) = 0.819, p = 0.369] is found in the speed of information processing between a group of chronic khat chewers and a non-chewing control group [68].

The importance of considering the use of other substances has been pronounced in the study of the neuropsychological impacts of khat use [69]. As compared to the khat-only group and the control group of non-users of khat and cigarettes, the concurrent khat and cigarette users recalled fewer words, had a slowed rate of verbal learning, and demonstrated delayed recall of previously learned verbal material [69]. The authors suggested that khat use alone may not affect immediate or delayed recall of previously learned words. Post-hoc tests did not show statistically significant differences in performance between non-users of khat and cigarettes, and khat-only users [69].

A study among chronic khat chewers shows that more deficits in neuropsychological functions are observed among khat chewers than among the non-chewing control group. The study suggests that the effects of khat are moderate and may not be noticeable after the consumption of a low dose [70].

G. Others mental health disorders and related conditions.

Under this section, those mental health disorders and related conditions mentioned in less than 5% of the studies are described. Among patients with schizophrenia, the use of khat has been associated with a lesser probability of properly following treatment for the disease. In a retrospective study to assess response to standard anti-psychotic treatment, a significant difference (P < 0.001) in the retention rate of the initial drug was observed among patients who use khat and patients who do not use khat (53.8% vs. 78.4%). Substituting the initial drug mainly due to lack of drug efficacy was significantly higher among moderate and heavy khat chewers than low and mild khat chewers (55% and 49.2% vs. 35.6% and 44.7% respectively, P < 0.001). It is hypothesized that khat could have hampered the response to initial antipsychotic drug treatment [71]. Concerning quality of life, the use of khat has been associated with a lower mental health component of quality-of-life scores [72–74]. Contrarily, among patients with schizophrenia, patients who use khat had higher scores in the mental health components of quality of life than patients who do not use khat (71.76 vs. 69.59) [75].

A study has shown that khat is believed to be related to suicidal death. In a case series study of khat-related deaths in the UK, four cases of suicide (three confirmed and one possible cases) due to psychoses caused and/ or worsened by long-term khat consumption have occurred [76]. Though lifetime khat use is associated with social phobia among college students in univariate analysis, the association was found to be insignificant in a multivariate regression model [77]. Khat chewing has been associated with sleeping disorders as a result of a pattern of heavy use [78]. Concerning mood disturbances, people who chew khat complain more frequently about negative affects than non-chewers [79]. People who chew khat are exposed to higher trait anger and negative responses during stress [80], and mild dysphoria and sedation after the end of the chewing sessions [81]. Studies among prisoners in correctional institutions show that inmates with psychopathy had three times higher odds of having a khat use history than those without psychopathy [46], and high-risk khat chewers were more likely to develop psychopathy than non-risk khat chewers, although the operational definition relating to what constitutes ‘risky use’ is not provided in the study [82]. Although khat use helps to cope with the side effects of antipsychotic drugs, drowsiness, and hunger among people with schizophrenia, it is also found to increase the risk of relapse and readmission to a psychiatric ward [20].

### Limitations of the studies

This review identified limitations related to study designs, several forms of biases, inability to control confounding variables, and sampling problems.

#### Design

As most studies employed a cross-sectional design, it is difficult to establish a cause-effect relationship.

#### Biases:

Most of the studies included in the review suffer from different forms of biases thematized under three topics: *social desirability bias*, *recall bias*, and *measurement bias*. Social desirability bias is due to administering questionnaires through face-to-face interviews and conducting studies in institutions like health facilities, correction centres, and schools. Due to the utilization of retrospective items in their questionnaires, several studies may have also been influenced by recall bias, which could impact the conclusions drawn about the association between khat use and mental health disorders. Many studies included in this review contain measurement biases as a result of (a) lack of an objective and standardized measure of khat use; (b) reliance on the lifetime khat use report (ever use of khat) to examine its association with mental disorders, which might not be the most sensitive indicator; (c) instruments employed in most instances to measure mental health disorders are general, and non-specific mental health disorders are reported; (d) most studies did not focus on the association between the level of khat use (e.g., frequency, amount, and age at first use) and mental disorders; and finally, (e) the studies utilized self-reports measurement for assessing khat chewing behavior and associated mental health problems, instead of a standard clinical interview or other objective measurements like urine or blood tests.

#### Confounding variables

The mediating role of genetic and neurodevelopmental factors, use of other substances, pesticides used by khat growing farmers, and environmental factors are not considered in many studies.

#### Study population and sampling

In some cases, results may not be generalizable since the studies used convenience sampling, and the institution-based nature of most studies also confines the generalizability of the findings.

## Discussion

This study presents a first attempt to systematically scope review the mental health disorders associated with khat chewing and to evaluate the strength of the available evidence. Most of the studies are from Ethiopia and this is to be expected given that Ethiopia is where the largest proportion of global khat production takes place [83] and probably holds the largest proportion of people who use khat globally. However, more studies are still needed from other traditional khat-using countries to comprehensively and comparatively understand the mental health effects of khat use and the modifying role of sociocultural and ecological factors.

Although studies assessing the association between khat use and mental health disorders have been published since 1981, a remarkable increase in the number of studies was observed only after 2011. This remarkable increase could be due to a recent dramatic upsurge in the prevalence of chewing [41, 84, 85], concerns about the mental health effects of khat and increasing number of researchers because of the expansion of higher education institutions in Ethiopia during this period.

The studies included in the scoping review are diverse in their designs, making difficult comparison among the studies and a meta-analysis of the results. The fact that most of the studies are cross-sectional limited the development of definite conclusions about the cause-effect relation, and interpretation of the results should be made with caution. As a result, further studies using robust methods like prospective cohort and experimental designs are needed. Such studies are required to record the start of the appearance of psychiatric symptoms and their relations to khat chewing, and to rule out or minimize the influences of confounding factors.

The majority of the studies did not employ standardized instruments or items to measure patterns of khat use. The instruments used vary considerably, from single-item question assessing the current or ever use of khat with yes/no or frequency of use as potential answers to multiple-item questionnaires (e.g., Severity of Dependence Scale (SDS) containing five items). However, more than two third of the studies measured khat use as a predictor variable with a single item ‘yes/no’ question. The use of a single item lifetime or current prevalence has a limitation since it groups together khat using people with very different consumptions patterns (in terms of frequency of use and amounts consumed) and with very different histories of use (e.g., in terms of length of the khat consumption career). Simply asking about lifetime or current prevalence also ignores the potential ‘dose-dependent’ effect of khat use on mental health. To overcome the measurement problems, future studies need to focus on assessing the levels of biomarkers (e.g., dopamine, serotonin, and cathinone) rather than purely relying on self-report of khat use. It should be noted, however, that such studies are resource intensive, and their realization needs greater investments. Moreover, specific measurement of khat use pattern (amount, frequency, duration, etc.) and its association with specific forms of mental disorders needs attention. Future studies should also focus on the dose-related effects of khat use on mental health, and to this end, standard scales with multiple items need to be employed to address the actual khat use experiences.

The standardized khat chewing measurement instruments used in a few studies are often adopted from studies of alcohol use disorders and measurements for disorders associated with other drugs. The use of such instruments could be less sensitive to screen khat-related disorders since they are not clinically tested and are not informed by qualitative studies of culture-specific khat use patterns and behaviors (for instance in the form of exploratory sequential designs). The reference periods used to measure khat chewing also considerably vary from last month use to life-time prevalence. Studies in other psychoactive substances, for instance alcohol, indicate that while the previous-month measures are better to avoid recall bias, the past year measures are preferred if capturing seasonal variability in consumption and measuring long term effects of consumption are needed [86]. In studies where the objective is to assess the mental health effects of khat chewing, thorough consideration of these pros and cons of different reference periods are needed. More importantly, whether the specific mental disorder being studied is better explained using which reference period needs consideration when designing a questionnaire.

About ten categories of mental disorders have been identified as being associated with the use of khat (see Table 3). Although findings are sometimes contradictory, most of the evidence associates khat use with mental health disorders. In general, ‘non-specific’ psychological distress is the most reported category. Four inconsistent findings have been reported in this category: khat is associated with greater risk of general psychological distress [47], there is no association between khat use and mental health disorders [48], frequent khat use is associated with general psychological distress whereas occasional use is not [34] and khat is used to treat psychological distress [53].

Although psychosis is the most studied specific mental health disorder in connection with khat use, most of the studies are case reports/ case series. Consequently, it proves challenging to extrapolate these findings to broader populations and establish definitive cause-and-effect relationships. Moreover, the majority of the studies are conducted on immigrants from traditionally khat-chewing regions, and it is not clear whether the development of psychosis is due to migration experiences or social isolation in destination countries. Very few studies used quantitative analysis of the connection between khat use and psychosis [26, 87–89]. A greater focus on the link between psychosis and khat use among migrants as compared to countries where khat is widely used could show the mediating role of traumas associated with migration or the weakening of khat chewing norms among chewers in the countries of destination.

Similarly, studies where depression, anxiety and/or stress are specifically assessed for their association with khat also presented conflicting findings. On the one hand, khat is considered as a risk factor [23, 54, 55, 57] and on the other hand insignificant associations are reported [40, 41, 90].

The study of ’khat use disorders’ is a topic where a better assessment of khat use patterns is made because of using standardized instruments like DAST [35] and DSM-5 [32]. The available evidence indicates that khat use disorders are more associated with daily chewing and greater quantity/frequency of khat use.

This review identified several biases, design problems, less generalizability of findings, and failure to control confounding variables. Keeping pace with the very fast-growing khat production and consumption [91], methodological advancement has not been made to study khat chewing’s impact on mental health. Nevertheless, arguments of a causal association between khat use and mental disorder have been used in the policy debate to prohibit the use of khat [27], leading several countries to criminalize the possession and chewing of khat. This in turn affects the personal and cultural right of khat chewing populations and the economy of subsistence, small scale farmers relying on khat farming.

Although this review has undergone a systematic search process of relevant studies in the major public health databases and relied on large volume of studies, every eligible study might not have been included. We recognize that our search strategy is limited as it does not cover individual mental health disorders, which often appear within the titles and abstracts of studies without mention of broader ‘mental health’. This was necessary to generate an achievable list of studies to analyze. We have tried to compensate for this omission by using hand searches to find studies missed in our database searching. However, we acknowledge that there may be studies still absent in our review despite meeting our eligibility criteria. Further reviews should aim to include both broad and specific mental health search terms within their search strategy to avoid this issue. Since this review also focused on publications in English, studies in other languages were excluded. Similar reviews in the future would benefit from including non-English studies.

## Conclusion

The review shows that the scientific interest in the study of khat uses’ effect on mental health has been increasing over the recent decades. However, this increasing interest is not supplemented with the employment of robust study designs capable of generating definitive conclusions about cause-effect relationships. Although most studies associate khat use with mental health disorders, they largely rely on cross-sectional study designs, which is prone to reverse causality, selection bias, and endogeneity problems. The lack of valid and standardized measurement tools for khat use has also limited our understanding of the mental health consequences of khat use. Given that the prevalence of khat use is increasing, there is a need for studies with better designs and instruments to document its mental health effects, and to determine which pattern of use may be problematic.

## Electronic supplementary material

Below is the link to the electronic supplementary material.

Supplementary Material 1

## Data Availability

All data generated or analyzed during this study are included in this published article [and its supplementary information files].

## References

[1] Odenwald M (2007). Chronic khat use and psychotic disorders: a review of the literature and future prospects. Sucht.

[2] Edwards B, Atkins N (2022). Exploring the association between khat use and psychiatric symptoms: a systematic review. BMJ open.

[3] Mekuriaw B et al. Prevalence of common mental disorder and its association with khat chewing among Ethiopian college students: a systematic review and meta-analysis. Psychiatry journal, 2020. 2020. 10.1155/2020/1462141 PMC6969648 31970194

[4] Astatkie A (2015). Prevalence of and factors associated with regular khat chewing among university students in Ethiopia. Subst abuse rehabilitation.

[5] Ihunwo A, Kayanja F, Amadi-Ihunwo U (2004). Use and perception of the psychostimulant, khat (catha edulis) among three occupational groups in south western Uganda. East Afr Med J.

[6] Odenwald M (2007). The consumption of khat and other drugs in somali combatants: a cross-sectional study. PLoS Med.

[7] Basunaid S, van Dongen M, Cleophas TJ (2008). Khat abuse in Yemen: a population-based survey. Clin Res Regul Affairs.

[8] Al-Hebshi N, Skaug N (2005). Khat (Catha edulis)—an updated review. Addict Biol.

[9] Reda AA et al. Prevalence and determinants of khat (Catha edulis) chewing among high school students in eastern Ethiopia: a cross-sectional study. PLoS ONE, 2012. 7(3). 10.1371/journal.pone.0033946 PMC3316517 22479484

[10] Al-Motarreb A, Baker K, Broadley KJ. Khat: pharmacological and medical aspects and its social use in Yemen. Phytotherapy Research: An International Journal devoted to pharmacological and toxicological evaluation of natural product derivatives, 2002. 16(5): p. 403–13. 10.1002/ptr.1106 12203257

[11] Balint EE, Falkay G, Balint GA (2009). Khat–a controversial plant. Wiener klinische Wochenschrift.

[12] Alemu WG et al. Prevalence and risk factors for khat use among youth students in Ethiopia: systematic review and meta-analysis, 2018. Annals of general psychiatry, 2020. 19(1): p. 1–10. 10.1186/s12991-020-00265-8 PMC7061479 32165908

[13] Nordgren J (2013). The moral entrepreneurship of anti-khat campaigners in Sweden–a critical discourse analysis. Drugs and Alcohol Today.

[14] Klein A, Beckerleg S, Hailu D (2009). Regulating khat—dilemmas and opportunities for the international drug control system. Int J Drug Policy.

[15] Odenwald M (2010). The stimulant khat—another door in the wall? A call for overcoming the barriers. J Ethnopharmacol.

[16] WHO Expert Committee on Drug Dependence, *WHO Expert Committee on Drug Dependence [meeting held in Geneva from 22 to 27 April 1985]: twenty-second report*. 1985: World Health Organization. 3938107

[17] Odenwald N, Lingenfelder B, Peschel W. Psychotic disorder, khat abuse and aggressive behavior in Somalia: a case report. Afr J Drug Alcohol Stud, 2008. 7(1).

[18] Odenwald M (2012). A pilot study on community-based outpatient treatment for patients with chronic psychotic disorders in Somalia: change in symptoms, functioning and co-morbid khat use. Int J Ment Health Syst.

[19] Teferra S (2011). Khat chewing in persons with severe mental illness in Ethiopia: a qualitative study exploring perspectives of patients and caregivers. Transcult Psychiatry.

[20] Bimerew M, Sonn F, Kortenbout W (2007). Substance abuse and the risk of readmission of people with schizophrenia at Amanuel Psychiatric Hospital. Ethiopia Curationis.

[21] Odenwald M (2005). Khat use as risk factor for psychotic disorders: a cross-sectional and case-control study in Somalia. BMC Med.

[22] Atnafie SA et al. Depression, anxiety, stress, and associated factors among khat chewers in Amhara Region, Northwest Ethiopia. Depress Res Treat, 2020. 2020. 10.1155/2020/7934892 PMC7533749 33062330

[23] Al Bahhawi T (2018). Depression, anxiety, and stress and their association with khat use: a cross-sectional study among Jazan University students, Saudi Arabia. Neuropsychiatr Dis Treat.

[24] Tesfaye E, Krahl W, Alemayehu S (2020). Khat induced psychotic disorder: case report. Subst Abuse Treat Prev Policy.

[25] Damena T, Mossie A, Tesfaye M (2011). Khat chewing and mental distress: a community based study, in jimma city, southwestern ethiopia. Ethiop J health Sci.

[26] Odenwald M (2009). Use of khat and posttraumatic stress disorder as risk factors for psychotic symptoms: a study of somali combatants. Soc Sci Med.

[27] Warfa N (2007). Khat use and mental illness: a critical review. Soc Sci Med.

[28] Numan N (2004). Exploration of adverse psychological symptoms in yemeni khat users by the symptoms Checklist-90 (SCL‐90). Addiction.

[29] Arksey H, O’Malley L (2005). Scoping studies: towards a methodological framework. Int J Soc Res Methodol.

[30] Peters MD (2015). Guidance for conducting systematic scoping reviews. JBI Evid Implement.

[31] Dhadphale M, Mengech A, Chege S (1981). MIRAA(Catha edulis) as a cause of psychosis. East Afr Med J.

[32] Duresso SW (2016). Is khat use disorder a valid diagnostic entity?. Addiction.

[33] Wolde A, Tesfaye Y, Yitayih Y. Psychopathy and Associated factors among newly admitted Prisoners in Correctional Institution located in Bench Sheko and West Omo Zone, South West Ethiopia: a cross-sectional study. Psychol Res Behav Manage, 2021: p. 261–73. 10.2147/PRBM.S294013 PMC7935445 33688279

[34] Gebrekidan Abbay A, Tibebe A, Mulatu H, Azadi (2018). Community knowledge, perceived beliefs and associated factors of mental distress: a case study from Northern Ethiopia. Int J Environ Res Public Health.

[35] Abdelwahab SI et al. Khat (Catha edulis Forsk.) dependence potential and pattern of use in Saudi Arabia. BioMed research international, 2015. 2015. 10.1155/2015/604526 PMC4561295 26380288

[36] El-Setouhy M et al. Khat dependency and psychophysical symptoms among chewers in Jazan Region, Kingdom of Saudi Arabia. BioMed research international, 2016. 2016. 10.1155/2016/2642506 PMC4789047 27022605

[37] Duresso SW (2018). Using the severity of dependence scale to screen for DSM-5 khat use disorder. Hum Psychopharmacology: Clin Experimental.

[38] Nakajima M, Hoffman R, Al’Absi M. Level of khat dependence, use patterns, and psychosocial correlates in Yemen: a cross-sectional investigation. EMHJ, 2017. 23(3). 10.26719/2017.23.3.161 28493262

[39] Alem A, Kebede D, Kullgren G (1999). The prevalence and socio-demographic correlates of khat chewing in Butajira, Ethiopia. Acta psychiatrica Scandinavica.

[40] Alsanosy RM, Mahfouz MS, Gaffar AM (2013). Khat chewing habit among school students of Jazan region, Saudi Arabia. PLoS ONE.

[41] Alsanosy RM, Mahfouz MS, Gaffar AM. Khat chewing among students of higher education in Jazan region, Saudi Arabia: prevalence, pattern, and related factors. BioMed research international, 2013. 2013. 10.1155/2013/487232 PMC3708399 23878809

[42] Mekuriaw B, Belayneh Z, Yitayih Y (2020). Magnitude of Khat use and associated factors among women attending antenatal care in Gedeo zone health centers, southern Ethiopia: a facility based cross sectional study. BMC Public Health.

[43] Nakajima M (2017). Correlates of khat use during pregnancy: a cross-sectional study. Addict Behav.

[44] Soboka M (2015). Khat use in people living with HIV: a facility-based cross-sectional survey from South West Ethiopia. BMC Psychiatry.

[45] Soboka M (2020). Magnitude and predictors of khat use among patients with tuberculosis in Southwest Ethiopia: a longitudinal study. PLoS ONE.

[46] Yitayih Y (2020). A cross-sectional study of psychopathy and khat abuse among prisoners in the correctional institution in Jimma. Ethiopia PloS one.

[47] Fekadu W (2015). Magnitude of Mental Illness and Associated factors among Holy Water users at Entoto St. Mary Church, Addis Ababa, Ethiopia, 2014. J Psychiatry.

[48] Soboka M, Gudina EK, Tesfaye M (2017). Psychological morbidity and substance use among patients with hypertension: a hospital-based cross-sectional survey from South West Ethiopia. Int J mental health Syst.

[49] Alem A, Kebede D, Kullgren GJAPS (1999). The prevalence and socio-demographic correlates of khat chewing in Butajira. Ethiopia.

[50] Dessie Y, Ebrahim J, Awoke T. Mental distress among university students in Ethiopia: a cross sectional survey. Pan Afr Med J, 2013. 15(1). 10.11604/pamj.2013.15.95.2173 PMC3810159 24198889

[51] Kerebih H, Ajaeb M, Hailesilassie H (2017). Common mental disorders among medical students in Jimma University, Southwest Ethiopia. Afr Health Sci.

[52] Dachew BA, Bisetegn TA, Berhe R, Gebremariam (2015). Prevalence of mental distress and associated factors among undergraduate students of University of Gondar, Northwest Ethiopia: a cross-sectional institutional based study. PLoS ONE.

[53] Douglas H, Boyle M, Lintzeris N (2011). The health impacts of khat: a qualitative study among Somali-Australians. Med J Aust.

[54] Deyessa N (2008). Depression among women in rural Ethiopia as related to socioeconomic factors: a community-based study on women in reproductive age groups. Scand J Public Health.

[55] Yeshaw Y, Mossie A (2017). Depression, anxiety, stress, and their associated factors among Jimma University staff, Jimma, Southwest Ethiopia, 2016: a cross-sectional study. Neuropsychiatr Dis Treat.

[56] Gebiresilus AG et al. Khat use prevalence, causes and its effect on mental health, Bahir-Dar, north west Ethiopia. Eur Sci J, 2014. 10(23).

[57] Bedaso A, Kediro G, Yeneabat T (2018). Factors associated with depression among prisoners in southern Ethiopia: a cross-sectional study. BMC Res Notes.

[58] Teshome Hambisa M, Derese A, Abdeta T. Depressive symptoms among Haramaya university students in Ethiopia: a cross-sectional study. Depression research and treatment, 2020. 2020. 10.1155/2020/5027918 PMC7013291 32099677

[59] Mains D, Hadley C, Tessema F (2013). Chewing over the future: khat consumption, anxiety, depression, and time among young men in Jimma, Ethiopia. Cult Med Psychiatry.

[60] Bultum JA (2018). Alcohol use disorder and associated factors among human immunodeficiency virus infected patients attending antiretroviral therapy clinic at Bishoftu General Hospital, Oromiya region, Ethiopia. PLoS ONE.

[61] Lemma A (2021). Alcohol use disorder and associated factors among University of Gondar undergraduate students: a cross-sectional study. J Subst Abuse Treat.

[62] Dawud B (2021). Substance Use Disorders and Associated factors among Adult Psychiatric Patients in Jimma Town, Southwest Ethiopia, 2017. Community-based cross-sectional study. Clin Med Insights: Psychiatry.

[63] Molla Z (2017). Tobacco dependence among people with mental illness: a facility-based cross sectional study from Southwest Ethiopia. BMC Res Notes.

[64] Dachew BA (2016). Suicidal thoughts among university students in Ethiopia. Ann Gen Psychiatry.

[65] Desalegn GT (2020). Suicide ideation, attempt, and determinants among medical students Northwest Ethiopia: an institution-based cross-sectional study. Ann Gen Psychiatry.

[66] Tilahun S. Prevalence and Associated Factors of Suicidal Behaviour among Postpartum Mother Attending at Public Health Centre, Addis Ababa, Ethiopia, 2021. 2021, Addis Ababa University: Unpublished thesis.

[67] Colzato LS (2011). Khat use is associated with impaired working memory and cognitive flexibility. PLoS ONE.

[68] Hoffman R, al’Absi M (2013). Working memory and speed of information processing in chronic khat users: preliminary findings. Eur Addict Res.

[69] Hoffman R, al’Absi M (2013). Concurrent use of khat and tobacco is associated with verbal learning and delayed recall deficits. Addiction.

[70] Ismail AA (2014). Neuropsychological functioning among chronic khat users in Jazan region, Saudi Arabia. Substance abuse.

[71] Hakami T et al. Effects of khat use on response to antipsychotic medications in patients with newly diagnosed schizophrenia: a retrospective study. East Mediterr Health J, 2021. 27(4). 10.26719/emhj.21.003 33955531

[72] Jaber AAS (2016). Evaluation of health-related quality of life among tuberculosis patients in two cities in Yemen. PLoS ONE.

[73] Sheikh KA (2014). Khat chewing and health related quality of life: cross-sectional study in Jazan region, Kingdom of Saudi Arabia. Health Qual Life Outcomes.

[74] Desalegn D, Girma S, Abdeta T (2020). Quality of life and its association with current substance use, medication non-adherence and clinical factors of people with schizophrenia in Southwest Ethiopia: a hospital-based cross-sectional study. Health Qual Life Outcomes.

[75] Yared M. *Determinants of health-related quality of life in patients with schizophrenia at Amanuel Mental Specialized Hospital, Addis Ababa, Ethiopia, Department of Psychiatry*. 2019, Addis Ababa University.

[76] Corkery JM (2011). Bundle of fun’or ‘bunch of problems’? Case series of khat-related deaths in the UK. Drugs: Educ Prev policy.

[77] Hajure M, Tariku M, Abdu Z. Prevalence and Associated factors of Social Phobia among College of Health Science Students, Mettu Town, Southwest Ethiopia 2019; institutional based cross-sectional study. The Open Public Health Journal, 2020. 13(1).

[78] Kennedy JG (1983). A medical evaluation of the use of qat in North Yemen. Soc Sci Med.

[79] Al’Absi M (2013). Effects of chronic khat use on cardiovascular, adrenocortical, and psychological responses to stress in men and women. Am J Addictions.

[80] Bongard S (2011). Khat use and trait anger: effects on affect regulation during an acute stressful challenge. Eur Addict Res.

[81] Nencini P, Ahmed AM, Elmi AS (1986). Subjective effects of khat chewing in humans. Drug Alcohol Depend.

[82] Wolde A, Tesfaye Y, Yitayih Y (2021). Psychopathy and Associated factors among newly admitted Prisoners in Correctional Institution located in Bench Sheko and West Omo Zone, South West Ethiopia: a cross-sectional study. Psychol Res Behav Manage.

[83] Cochrane L, O’Regan D (2016). Legal harvest and illegal trade: Trends, challenges, and options in khat production in Ethiopia. Int J Drug Policy.

[84] Gebrehanna E, Berhane Y, Worku A (2014). Khat chewing among Ethiopian University Students-a growing concern. BMC Public Health.

[85] Yitayih Y, van Os J (2021). Prevalence and determinants of chewing khat among women in Ethiopia: data from ethiopian demographic and health survey 2016. BMC Psychiatry.

[86] Greenfield TK, Kerr WC (2008). Alcohol measurement methodology in epidemiology: recent advances and opportunities. Addiction.

[87] Adorjan K (2017). Khat use and occurrence of psychotic symptoms in the general male population in Southwestern Ethiopia: evidence for sensitization by traumatic experiences. World Psychiatry.

[88] Ongeri L (2019). Khat use and psychotic symptoms in a rural Khat growing population in Kenya: a household survey. BMC Psychiatry.

[89] Widmann M (2014). Khat use, PTSD and psychotic symptoms among somali refugees in Nairobi–a pilot study. Front public health.

[90] Mains D, Hadley C, Tessema F (2013). Chewing over the future: khat consumption, anxiety, depression, and time among young men in Jimma. Ethiopia Cult Med Psychiatry.

[91] Rather RA (2021). Prevalence of Khat (Catha edulis) chewing and its determinants: a respondent-driven survey from Hossana, Ethiopia. Subst Abuse Rehabilitation.

