# Supplementary material for: Is chewing khat associated with mental health disorders? A scoping review of the content and quality of the current evidence base
Source: Subst Abuse Treat Prev Policy. 2023 Jun 27;18:39. doi: 10.1186/s13011-023-00545-y (PMC10303364; doi:10.1186/s13011-023-00545-y)
Supplement: Supplementary file 1 — Supplementary Material 1 [file 13011_2023_545_MOESM1_ESM.docx]

**Supplementary 1: List of the included studies (1-108)**

1. Abdillahi FA, Ismail EA, Singh SP. Mental health in Somaliland: a critical situation. BJPsych international. 2020;17(1):11-4.

2. Adraro W, Kerebih H, Tesema W, Abamecha F, Hailesilassie H. Nearly three in every five prisoners experience common mental disorders (CMDs) in Jimma correctional institution; south-West Ethiopia. BMC public health. 2019;19:1-9.

3. Alem A, Kebede D, Kullgren G. The prevalence and socio‐demographic correlates of khat chewing in Butajira, Ethiopia. Acta Psychiatrica Scandinavica. 1999;100:84-91.

4. Alenko A, Girma S, Abera M, Workicho A. Children emotional and behavioural problems and its association with maternal depression in Jimma town, southwest Ethiopia. General psychiatry. 2020;33(4).

5. Alexander J, Staugas R, El-Domeiri O. Khat concerns in Australia: hyperbole or understated? Australian and New Zealand Journal of Psychiatry. 2010;44(10):960-1.

6. Alsanusy R, El-Setouhy M. Why would khat chewers quit? An in-depth, qualitative study on Saudi Khat quitters. Substance abuse. 2013;34(4):389-95.

7. Bongard S, Nakajima M. Khat chewing and acculturation in East-African migrants living in Frankfurt am Main/Germany. Journal of ethnopharmacology. 2015;164:223-8.

8. Chaka A, Awoke T, Yohannis Z, Ayano G, Tareke M, Abate A, et al. Determinants of depression among people with epilepsy in Central Ethiopia. Annals of general psychiatry. 2018;17:1-7.

9. Colzato LS, Ruiz MJ, van den Wildenberg WP, Hommel B. Khat use is associated with increased response conflict in humans. Human Psychopharmacology: Clinical and Experimental. 2012;27(3):315-21.

10. Colzato LS, Sellaro R, Ruiz MJ, Sikora K, Hommel B. Acute khat use reduces response conflict in habitual users. Frontiers in Human Neuroscience. 2013;7:285.

11. Critchlow S, Seifert R. Khat-induced paranoid psychosis. The British Journal of Psychiatry. 1987;150(2):247-9.

12. Cumming C, Butt J, Hersi A, Tohow A, Young J. Khat use and perceived health problems among African migrants in Australia: an exploratory study. Eastern Mediterranean Health Journal. 2021;27(5):491-500.

13. Dachew BA, Azale Bisetegn T, Berhe Gebremariam R. Prevalence of mental distress and associated factors among undergraduate students of University of Gondar, Northwest Ethiopia: a cross-sectional institutional based study. Plos one. 2015;10(3):e0119464.

14. Daud AH, Saleem S, Mahmood Z. DIFFERENTIAL EFFECTIVENESS OF COGNITIVE BEHAVIOR THERAPY AND PSYCHO EDUCATION THERAPY ON KHAT CHEWING BEHAVIOR AND ASSOCIATED MENTAL HEALTH PROBLEMS. Journal of Postgraduate Medical Institute. 2019;33(4).

15. Dhadphale M, Omolo O. Psychiatric morbidity among khat chewers. East Afr Med J. 1988;65(6):355-9.

16. Eticha T, Teklu A, Ali D, Solomon G, Alemayehu A. Factors associated with medication adherence among patients with schizophrenia in Mekelle, Northern Ethiopia. PloS one. 2015;10(3):e0120560.

17. Gelaye B, Okeiga J, Ayantoye I, Berhane HY, Berhane Y, Williams MA. Association of suicidal ideation with poor sleep quality among Ethiopian adults. Sleep and Breathing. 2016;20:1319-26.

18. James Giannini A, Castellani S. A manic-like psychosis due to Khat Catha edulis Forsk. Journal of Toxicology: Clinical Toxicology. 1982;19(5):455-9.

19. Gizachew KD, Chekol YA, Basha EA, Mamuye SA, Wubetu AD. Suicidal ideation and attempt among people living with HIV/AIDS in selected public hospitals: Central Ethiopia. Annals of general psychiatry. 2021;20(1):1-18.

20. Gough S, Cookson I, Mayberry J, Morgan G, Perkin E. Khat-induced schizophreniform psychosis in UK. The Lancet. 1984;323(8374):455.

21. Granek M, Shalev A, Weingarten A. Khat‐induced hypnagogic hallucinations. Acta Psychiatrica Scandinavica. 1988;78(4):458-61.

22. Griffiths P, Gossop M, Wickenden S, Dunworth J, Harris K, Lloyd C. A transcultural pattern of drug use: qat (khat) in the UK. The British Journal of Psychiatry. 1997;170(3):281-4.

23. Hajure M, Dibaba B, Shemsu S, Desalegn D, Reshad M, Mohammedhussein M. Psychological distress among health care workers in health facilities of mettu town during COVID-19 outbreak, South West Ethiopia, 2020. Frontiers in Psychiatry. 2021;12:574671.

24. Hajure M, Tariku M, Abdu Z. Prevalence and associated factors of social phobia among college of health science students, Mettu town, southwest Ethiopia 2019; institutional based cross-sectional study. The Open Public Health Journal. 2020;13(1).

25. Hakami T, Mahmoud M, Mohammed B, El-Setouhy M. Effects of khat use on response to antipsychotic medications in patients with newly diagnosed schizophrenia: a retrospective study. Eastern Mediterranean Health Journal. 2021;27(4):353-60.

26. Hambisa S, Siraj J, Mesafint G, Yimam M. Assessment of psychological distress and associated factors among hospitalized patients during the COVID-19 pandemic at selected hospitals in Southwest Ethiopia. Neuropsychiatric Disease and Treatment. 2021:885-92.

27. Hassan NA, Gunaid AA, El-Khally FM, Murray-Lyon IM. The effect of chewing Khat leaves on human mood. Neurosciences Journal. 2002;7(3):184-7.

28. Hersi L, Tesfay K, Gesesew H, Krahl W, Ereg D, Tesfaye M. Mental distress and associated factors among undergraduate students at the University of Hargeisa, Somaliland: a cross-sectional study. International journal of mental health systems. 2017;11(1):1-8.

29. Hoffman R, al'Absi M. Concurrent use of khat and tobacco is associated with verbal learning and delayed recall deficits. Addiction. 2013;108(10):1855-62.

30. Hoffman R, al’Absi M. Working memory and speed of information processing in chronic khat users: preliminary findings. European Addiction Research. 2013;19(1):1-6.

31. Hunduma G, Girma M, Digaffe T, Weldegebreal F, Tola A. Prevalence and determinants of common mental illness among adult residents of Harari Regional State, Eastern Ethiopia. Pan African Medical Journal. 2017;28(1).

32. Ismail AA, El Sanosy RM, Rohlman DS, El-Setouhy M. Neuropsychological functioning among chronic khat users in Jazan region, Saudi Arabia. Substance abuse. 2014;35(3):235-44.

33. Jaber AAS, Khan AH, Syed Sulaiman SA, Ahmad N, Anaam MS. Evaluation of health-related quality of life among tuberculosis patients in two cities in Yemen. PloS one. 2016;11(6):e0156258.

34. Jager AD, Sireling L. Natural history of khat psychosis. Australian and New Zealand Journal of Psychiatry. 1994;28(2):331-2.

35. Kassim S, Croucher R, Al'Absi M. Khat dependence syndrome: a cross sectional preliminary evaluation amongst UK-resident Yemeni khat chewers. Journal of ethnopharmacology. 2013;146(3):835-41.

36. Kennedy JG, Teague J, Rokaw W, Cooney E. A medical evaluation of the use of qat in North Yemen. Social Science & Medicine. 1983;17(12):783-93.

37. Kerebih H, Ajaeb M, Hailesilassie H. Common mental disorders among medical students in Jimma University, Southwest Ethiopia. African health sciences. 2017;17(3):844-51.

38. Lemieux AM, Nakajima M, Saif-Ali R, Al-Habori M, Dokam A, al'Absi M. Anger, anxiety, and depressive affect as predictors of stress-induced cortisol production in khat and tobacco users. Addictive Behaviors. 2018;82:195-201.

39. Lemma A, Salelew E, Demilew D, Tesfaye W, Shumet S, Kerebih H. Alcohol use disorder and associated factors among University of Gondar undergraduate students: A cross-sectional study. Journal of Substance Abuse Treatment. 2021;129:108373.

40. Mains D, Hadley C, Tessema F. Chewing over the future: khat consumption, anxiety, depression, and time among young men in Jimma, Ethiopia. Culture, Medicine, and Psychiatry. 2013;37:111-30.

41. McLaren P. Khat psychosis. The British Journal of Psychiatry. 1987;150(5):712-3.

42. Mekuriaw B, Belayneh Z, Yitayih Y. Magnitude of Khat use and associated factors among women attending antenatal care in Gedeo zone health centers, southern Ethiopia: a facility based cross sectional study. BMC Public Health. 2020;20:1-8.

43. Molla Z, Dube L, Krahl W, Soboka M. Tobacco dependence among people with mental illness: a facility-based cross sectional study from Southwest Ethiopia. BMC research notes. 2017;10(1):1-7.

44. Mukherjee TI, Hirsch-Moverman Y, Saito S, Gadisa T, Melaku Z, Howard AA. Determinants of alcohol use among people living with HIV initiating isoniazid preventive therapy in Ethiopia. Drug and alcohol dependence. 2019;204:107465.

45. Nakajima M, al’Absi M. Influences of fasting on stress response and withdrawal symptoms in habitual Khat users. European addiction research. 2021;27(1):49-57.

46. Nakajima M, Jebena MG, Taha M, Tesfaye M, Gudina E, Lemieux A, et al. Correlates of khat use during pregnancy: a cross-sectional study. Addictive behaviors. 2017;73:178-84.

47. Nencini P, Ahmed AM, Elmi AS. Subjective effects of khat chewing in humans. Drug and alcohol dependence. 1986;18(1):97-105.

48. Nielen RJ, Heijden FMvd, Tuinier S, Verhoeven WM. Khat and mushrooms associated with psychosis. The World Journal of Biological Psychiatry. 2004;5(1):49-53.

49. Njuguna J, Olieva S, Muruka C, Owek C. Khat consumption in Masalani town, northeastern Kenya. Journal of Psychoactive Drugs. 2013;45(4):355-9.

50. Numan N. Exploration of adverse psychological symptoms in Yemeni khat users by the Symptoms Checklist‐90 (SCL‐90). Addiction. 2004;99(1):61-5.

51. Odenwald M, Hinkel H, Schauer E, Schauer M, Elbert T, Neuner F, et al. Use of khat and posttraumatic stress disorder as risk factors for psychotic symptoms: a study of Somali combatants. Social science & medicine. 2009;69(7):1040-8.

52. Odenwald M, Neuner F, Schauer M, Elbert T, Catani C, Lingenfelder B, et al. Khat use as risk factor for psychotic disorders: a cross-sectional and case-control study in Somalia. BMC medicine. 2005;3(1):1-10.

53. Ongeri L, Kirui F, Muniu E, Manduku V, Kirumbi L, Atwoli L, et al. Khat use and psychotic symptoms in a rural Khat growing population in Kenya: a household survey. BMC psychiatry. 2019;19(1):1-10.

54. Sheikh KA, El-Setouhy M, Yagoub U, Alsanosy R, Ahmed Z. Khat chewing and health related quality of life: cross-sectional study in Jazan region, Kingdom of Saudi Arabia. Health and quality of life outcomes. 2014;12:1-10.

55. Soboka M, Gudina EK, Tesfaye M. Psychological morbidity and substance use among patients with hypertension: a hospital-based cross-sectional survey from South West Ethiopia. International Journal of Mental Health Systems. 2017;11:1-7.

56. Soboka M, Tesfaye M, Feyissa GT, Hanlon C. Khat use in people living with HIV: a facility-based cross-sectional survey from South West Ethiopia. BMC psychiatry. 2015;15(1):1-7.

57. Soboka M, Tolessa O, Tesfaye M, Adorjan K, Krahl W, Tesfaye E, et al. Magnitude and predictors of khat use among patients with tuberculosis in Southwest Ethiopia: A longitudinal study. Plos one. 2020;15(7):e0236154.

58. Jini D, Tariku G, Zerihun A, Bisrat Z, Adissu G. Mental distress and its associated factors among students of Mizan Aman Health Science College, Ethiopia. J Med Sci. 2017;17(2):61-7.

59. Teferra S, Hanlon C, Alem A, Jacobsson L, Shibre T. Khat chewing in persons with severe mental illness in Ethiopia: a qualitative study exploring perspectives of patients and caregivers. Transcultural psychiatry. 2011;48(4):455-72.

60. Teferra S, Shibre T. Perceived causes of severe mental disturbance and preferred interventions by the Borana semi-nomadic population in southern Ethiopia: a qualitative study. BMC psychiatry. 2012;12:1-9.

61. Tesfaye E, Krahl W, Alemayehu S. Khat induced psychotic disorder: case report. Substance abuse treatment, prevention, and policy. 2020;15(1):1-5.

62. Tesfaye Kelemu R, Bayray Kahsay A, Ahmed KY. Prevalence of mental distress and associated factors among Samara university students, northeast Ethiopia. Depression research and treatment. 2020;2020.

63. Teshome Hambisa M, Derese A, Abdeta T. Depressive symptoms among Haramaya university students in Ethiopia: a cross-sectional study. Depression research and treatment. 2020;2020.

64. Tilahun H, Awoke N, Geda B, Mesfin F. Depression and associated factors among adult inpatients at public hospitals of Harari regional state, eastern Ethiopia. Psychiatry Journal. 2018;2018.

65. Toennes SW, Harder S, Schramm M, Niess C, Kauert GF. Pharmacokinetics of cathinone, cathine and norephedrine after the chewing of khat leaves. British journal of clinical pharmacology. 2003;56(1):125-30.

66. Toennes SW, Kauert GF. Driving under the influence of khat—alkaloid concentrations and observations in forensic cases. Forensic science international. 2004;140(1):85-90.

67. Tulloch AD, Frayn E, Craig TK, Nicholson TR. Khat use among Somali mental health service users in South London. Social psychiatry and psychiatric epidemiology. 2012;47:1649-56.

68. Widmann M, Warsame AH, Mikulica J, von Beust J, Isse MM, Ndetei D, et al. Khat use, PTSD and psychotic symptoms among Somali refugees in Nairobi–a pilot study. Frontiers in public health. 2014;2:71.

69. Wolde A, Tesfaye Y, Yitayih Y. Psychopathy and Associated Factors Among Newly Admitted Prisoners in Correctional Institution Located in Bench Sheko and West Omo Zone, South West Ethiopia: A Cross-Sectional Study. Psychology Research and Behavior Management. 2021:261-73.

70. Wolf KM, Zoucha R, McFarland M, Salman K, Dagne A, Hashi N. Somali immigrant perceptions of mental health and illness: An ethnonursing study. Journal of transcultural nursing. 2016;27(4):349-58.

71. Yeshaw Y, Mossie A. Depression, anxiety, stress, and their associated factors among Jimma University staff, Jimma, Southwest Ethiopia, 2016: a cross-sectional study. Neuropsychiatric disease and treatment. 2017:2803-12.

72. Yitayih Y, Soboka M, Tesfaye E, Abera M, Mamaru A, Adorjan K. Trauma exposure and alcohol use disorder among prisoners in Jimma Zone correctional institution, Southwest Ethiopia: a cross-sectional study. BMC Research notes. 2019;12(1):1-6.

73. Yitayih Y, Soboka M, Tesfaye E, Abera M, Mamaru A, Adorjan K. A cross-sectional study of psychopathy and khat abuse among prisoners in the correctional institution in Jimma, Ethiopia. PloS one. 2020;15(1):e0227405.

74. Young JT, Butt J, Hersi A, Tohow A, Mohamed DH. Khat dependence, use patterns, and health consequences in Australia: an exploratory study. Journal of studies on alcohol and drugs. 2016;77(2):343-8.

75. Getachew F. Effect of Chronic Khat Chewing on Mental Health, Blood Pressure and Pulse Rate in Hosanna Town, Ethiopia: Addis Ababa University; 2016.

76. Gebiresilus AG, Gebresilus BG, Yizengaw SS, Sewasew DT, Mengesha TZ. Khat use prevalence, causes and its effect on mental health, Bahir-Dar, north west Ethiopia. European Scientific Journal. 2014;10(23).

77. Yared T. Determinants of health-related quality of life in patients with schizophrenia at Amanuel Mental Specialized Hospital, Addis Ababa, Ethiopia

Addis Ababa: Addis Ababa University; 2019.

78. Abdelwahab SI, Alsanosy RM, Rahim B-eE, Mohan S, Taha S, Mohamed Elhassan M, et al. Khat (Catha edulis Forsk.) dependence potential and pattern of use in Saudi Arabia. BioMed research international. 2015;2015.

79. Adorjan K, Odenwald M, Widmann M, Tesfaye M, Tessema F, Toennes S, et al. Khat use and occurrence of psychotic symptoms in the general male population in Southwestern Ethiopia: evidence for sensitization by traumatic experiences. World Psychiatry. 2017;16(3):323.

80. Al'Absi M, Khalil NS, Al Habori M, Hoffman R, Fujiwara K, Wittmers L. Effects of chronic khat use on cardiovascular, adrenocortical, and psychological responses to stress in men and women. The American Journal on Addictions. 2013;22(2):99-107.

81. Bahhawi TA, Albasheer OB, Makeen AM, Arishi AM, Hakami OM, Maashi SM, et al. Depression, anxiety, and stress and their association with khat use: a cross-sectional study among Jazan University students, Saudi Arabia. Neuropsychiatric Disease and Treatment. 2018:2755-61.

82. Gebrekidan Abbay A, Tibebe Mulatu A, Azadi H. Community knowledge, perceived beliefs and associated factors of mental distress: a case study from Northern Ethiopia. International Journal of Environmental Research and Public Health. 2018;15(11):2423.

83. Alsanosy RM, Mahfouz MS, Gaffar AM. Khat chewing habit among school students of Jazan region, Saudi Arabia. Plos one. 2013;8(6):e65504.

84. Alsanosy RM, Mahfouz MS, Gaffar AM. Khat chewing among students of higher education in Jazan region, Saudi Arabia: prevalence, pattern, and related factors. BioMed research international. 2013;2013.

85. Atnafie SA, Muluneh NY, Getahun KA, Woredekal AT, Kahaliw W. Depression, anxiety, stress, and associated factors among khat chewers in Amhara region, Northwest Ethiopia. Depression research and treatment. 2020;2020:1-12.

86. Bedaso A, Kediro G, Yeneabat T. Factors associated with depression among prisoners in southern Ethiopia: a cross-sectional study. BMC research notes. 2018;11:1-6.

87. Bimerew M, Sonn F, Kortenbout W. Substance abuse and the risk of readmission of people with schizophrenia at Amanuel Psychiatric Hospital, Ethiopia. Curationis. 2007;30(2):74-81.

88. Bongard S, al’Absi M, Khalil NS, Al Habori M. Khat use and trait anger: effects on affect regulation during an acute stressful challenge. European Addiction Research. 2011;17(6):285-91.

89. Bultum JA, Yigzaw N, Demeke W, Alemayehu M. Alcohol use disorder and associated factors among human immunodeficiency virus infected patients attending antiretroviral therapy clinic at Bishoftu General Hospital, Oromiya region, Ethiopia. PloS one. 2018;13(3):e0189312.

90. Colzato LS, Ruiz MJ, van den Wildenberg WP, Hommel B. Khat use is associated with impaired working memory and cognitive flexibility. PloS one. 2011;6(6):e20602.

91. Corkery JM, Schifano F, Oyefeso A, Ghodse AH, Tonia T, Naidoo V, et al. ‘Bundle of fun’or ‘bunch of problems’? Case series of khat-related deaths in the UK. Drugs: education, prevention and policy. 2011;18(6):408-25.

92. Dachew BA, Bifftu BB, Tiruneh BT, Anlay DZ, Wassie MA. Suicidal thoughts among university students in Ethiopia. Annals of General Psychiatry. 2016;17(1):1-5.

93. Damena T, Mossie A, Tesfaye M. Khat chewing and mental distress: a community based study, in jimma city, southwestern ethiopia. Ethiopian journal of health sciences. 2011;21(1):37-46.

94. Dawud B, Yeshigeta E, Negash A, Mamaru A, Agenagnew L, Tolosa D, et al. substance use disorders and associated factors among adult psychiatric patients in Jimma Town, Southwest Ethiopia, 2017. Community-based cross-sectional study. Clinical Medicine Insights: Psychiatry. 2021;12:1179557321989699.

95. Desalegn D, Girma S, Abdeta T. Quality of life and its association with current substance use, medication non-adherence and clinical factors of people with schizophrenia in Southwest Ethiopia: a hospital-based cross-sectional study. Health and Quality of Life Outcomes. 2020;18:1-9.

96. Desalegn GT, Wondie M, Dereje S, Addisu A. Suicide ideation, attempt, and determinants among medical students Northwest Ethiopia: an institution-based cross-sectional study. Annals of general psychiatry. 2020;19(1):1-8.

97. Dessie Y, Ebrahim J, Awoke T. Mental distress among university students in Ethiopia: a cross sectional survey. Pan African Medical Journal. 2013;15(1).

98. Deyessa N, Berhane Y, Alem A, Hogberg U, Kullgren G. Depression among women in rural Ethiopia as related to socioeconomic factors: a community-based study on women in reproductive age groups. Scandinavian journal of public health. 2008;36(6):589-97.

99. Dhadphale M, Mengech A, Chege S. MIRAA(Catha edulis) as a Cause of Psychosis. East African Medical Journal. 1981;58(2):130-5.

100. Douglas H, Boyle M, Lintzeris N. The health impacts of khat: a qualitative study among Somali‐Australians. Medical Journal of Australia. 2011;195(11-12):666-9.

101. Duresso SW, Matthews AJ, Ferguson SG, Bruno R. Is khat use disorder a valid diagnostic entity? Addiction. 2016;111(9):1666-76.

102. Duresso SW, Matthews AJ, Ferguson SG, Bruno R. Using the Severity of Dependence Scale to screen for DSM‐5 khat use disorder. Human Psychopharmacology: Clinical and Experimental. 2018;33(2):e2653.

103. El-Setouhy M, Alsanosy RM, Alsharqi A, Ismail AA. Khat dependency and psychophysical symptoms among chewers in Jazan Region, Kingdom of Saudi Arabia. BioMed research international. 2016;2016.

104. Fekadu W, Haregwoin M, Kibrom H. Magnitude of mental illness and associated factors among Holy water users at Entoto ST Mary church, Addis Ababa, Ethiopia, 2014. J Psychiatry. 2014;18:285.

105. Nakajima M, Hoffman R, Al’Absi M. Level of khat dependence, use patterns, and psychosocial correlates in Yemen: a cross-sectional investigation. EMHJ. 1995;23(3).

106. Kiros E. The Perceived Reasons and Effects of Khat Abuse: The case of Selected Rehabilitation Centers in Addis Ababa. Addis Ababa: Addis Ababa Unversity; 2019.

107. Getachew F. Effect of Chronic Khat Chewing on Mental Health, Blood Pressure and Pulse Rate in Hosanna Town, Ethiopia. Addis Ababa: Addis Ababa University; 2016.

108. Tilahun S. Prevalence and Associated Factors of Suicidal Behaviour among Postpartum Mother Attending at Public Health Centre, Addis Ababa, Ethiopia, 2021. Addis Ababa: Addis Ababa University; 2021.
